# Sternal plating for primary and secondary sternal closure; can it improve sternal stability?

**DOI:** 10.1186/1749-8090-4-19

**Published:** 2009-05-07

**Authors:** Hosam Fawzy, Nasser Alhodaib, C David Mazer, Alana Harrington, David Latter, Daniel Bonneau, Lee Errett, James Mahoney

**Affiliations:** 1 Division of Cardiovascular and Thoracic Surgery, Department of Surgery, Terrence Donnelly Heart Center, Keenan Research Center in the Li Ka Shing Knowledge Institute of St Michael's Hospital, University of Toronto, 30 Bond Street, Toronto, Ontario, M5B 1W8, Canada; 2 Division of Plastic Surgery, Department of Surgery, Terrence Donnelly Heart Center, Keenan Research Center in the Li Ka Shing Knowledge Institute of St Michael's Hospital, University of Toronto, 30 Bond Street, Toronto, Ontario, M5B 1W8, Canada; 3 Anesthesia and Critical Care, Terrence Donnelly Heart Center, Keenan Research Center in the Li Ka Shing Knowledge Institute of St Michael's Hospital, University of Toronto, 30 Bond Street, Toronto, Ontario, M5B 1W8, Canada

## Abstract

**Background:**

Sternal instability with mediastinitis is a very serious complication after median sternotomy. Biomechanical studies have suggested superiority of rigid plate fixation over wire cerclage for sternal fixation. This study tests the hypothesis that sternal closure stability can be improved by adding plate fixation in a human cadaver model.

**Methods:**

Midline sternotomy was performed in 18 human cadavers. Four sternal closure techniques were tested: (1) approximation with six interrupted steel wires; (2) approximation with six interrupted cables; (3) closure 1 (wires) or 2 (cables) reinforced with a transverse sternal plate at the sixth rib; (4) Closure using 4 sternal plates alone. Intrathoracic pressure was increased in all techniques while sternal separation was measured by three pairs of sonomicrometry crystals fixed at the upper, middle and lower parts of the sternum until 2.0 mm separation was detected. Differences in displacement pressures were analyzed using repeated measures ANOVA and Regression Coefficients.

**Results:**

Intrathoracic pressure required to cause 2.0 mm separation increased significantly from 183.3 ± 123.9 to 301.4 ± 204.5 in wires/cables alone vs. wires/cables plus one plate respectively, and to 355.0 ± 210.4 in the 4 plates group (p < 0.05). Regression Coefficients (95% CI) were 120 (47–194) and 142 (66–219) respectively for the plate groups.

**Conclusion:**

Transverse sternal plating with 1 or 4 plates significantly improves sternal stability closure in human cadaver model. Adding a single sternal plate to primary closure improves the strength of sternal closure with traditional wiring potentially reducing the risk of sternal dehiscence and could be considered in high risk patients.

## Introduction

Open heart surgery is one of the most common procedures done in North America. It is usually performed through the median sternotomy that was re-introduced by Julian in 1957 [1]. Median sternotomy can be performed quickly, provides excellent exposure of the heart, which facilitates different cardiac procedures, and is well tolerated by most patients [2]. The current standard technique for sternal closure remains the cerclage stainless steel wires.

Complications from median sternotomy occur in approximately 0.3 – 5% of cases and is associated with significant morbidity and mortality [3]. Loosening and failure of sternal fixation associated with wound dehiscence have major and minor sternal healing complications. The wire closure technique under normal physiologic loads can lead to inadequate fixation and sternal dehiscence [4]. Loosening of this technique occurs when sternal stresses concentrated at the steel wire cause it to cut into the bone, allowing variable degrees of motion to occur. Off center sternotomy, osteoporosis and strong mechanical forces such as with coughing contribute to the inability of wire closure to maintain fixation, leading to acute separation or in the long-term sternal nonunion. Infection is also associated with loosening. Wound dehiscence, purulent drainage, and sternal instability are indicative of deep sternal wound infection and mediastinitis. A vicious circle is created by wound dehiscence, sternal movement and infection which only can be treated by surgical treatment including wound debridement. In addition to a variety of wound management techniques, effective rigid closure of the sternum that restores stability, promoting primary healing of the sternum can be considered. Numerous methods for sternal fixation have been described to treat sternal dehiscence but there is no ideal method yet [5-8]. To analyze the effectiveness of plating to maintain sternal stability, we evaluated the present standard technique of wiring and compared it to a new potentially more rigid sternal fixation system. This study tests the hypothesis that sternal closure stability can be improved by sternal plating, which distributes the force applied to the plated sternum over a larger area. The aim of this study is to determine whether the sternal fixation system improves the mechanical stability of sternal closure using four different methods of primary sternal closure.

## Materials and methods

**Figure 1:**
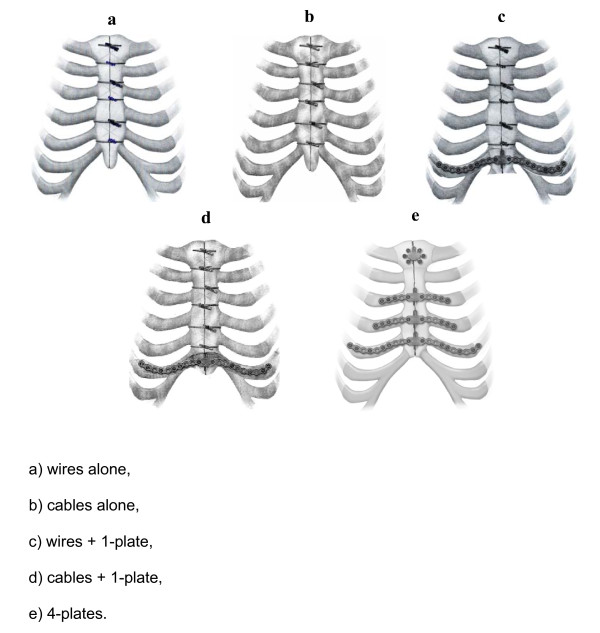
**Pictorial representation of the five different sternal closure techniques used**. (a) Approximation with stainless steel sternal wires alone. (b) Approximation with multifilament sternal cables alone.(c) Approximation with sternal wires and a single plate at rib number 6. (d) Approximation with sternal cables and a single plate at rib number 6. (e) Approximation with four plates.

With approval of the St. Michael's Hospital Research Ethics Board and the University of Toronto, midline sternotomy was performed in 18 adult human cadavers. Each cadaver was subject to three sternal closure techniques in three subsequent stages as follows (Figure 1a–e):

1. Approximation with stainless steel sternal wires alone (n = 9) or multifilament sternal cables alone (n = 9)

2. Retightening of the initial fixation as in step 1 and application of a single plate at rib number 6.

3. Removal of wires/cables and plate followed by fixation with a transverse sternal plate system with 4 plates (n = 18).

Nine cadavers were initially closed with six standard stainless steel wires (size no.6) in a simple interrupted fashion, two around the manubrium and four around the sternum at the 2^nd ^through 5^th ^intercostal spaces. The wires were symmetrically tightened by twisting with a large needle driver. After stability testing (see below), the wires were retightened and a single transverse locking sternal plate (4 screws/side; Synthes Titanium Sternal Fixation System, Synthes CMF, West Chester, PA, USA) was placed at rib number 6 and the fixation was retested. Finally, all wires and the plate were removed and the sternum was fixed with four transverse plates; one at the manubrium and three straight plates fixed transversely rib-to-rib from ribs 3–5.

The other nine cadavers were subjected to initial sternal closure with 1.0 mm multifilament stainless steel cables (Synthes Sternal Cables, Synthes CMF, West Chester, Pennsylvannia, USA) with subsequent plate closure in the same sequence as the wires group. The cables were tightened using the cable tightener as per the manufacturer's specifications.

The sequence of fixation in all cadavers was selected to minimize any effects of the previous closure technique and allow comparison of different techniques studied.

Following completion of each closure technique intrathoracic pressure was increased while the separation between the two halves of the sternum was continuously measured. The amount of pressure needed to induce sternal distraction was compared for all closure methods. The primary outcome was the intrathoracic pressure required to separate the sternal edges by 2.0 mm. This endpoint was chosen as an amount of sternal separation that would be clinically important and to avoid significant damage of the sternal bone integrity for further testing with the subsequent closure techniques.

### Preparation of the cadavers

The median sternotomy was performed using a standard pneumatically powered reciprocating sternal saw (Stryker, Kalamazoo MI). An inflatable rubber bladder was seated immediately beneath the sternum. A calibrated pressure transducer (Utah Medical Products, Midvale, Utah, USA) was connected to the inflatable bladder and used to record the pressure within the closed system at appropriate range and intervals. The system was configured to continuously monitor real-time intrathoracic pressure and intercrystal distance while simultaneously recording absolute values every two milliseconds using a computerized data-acquisition system (Power Lab, AD Instruments, Colorado Springs, Colorado, USA).

### Measurement of sternal separation

Three pairs of 5 MHz piezoelectric crystals were used to measure distraction of the sternal closure site using sonomicrometry (Sonometrics, Triton Technology). The crystals were fixed to the ventral edge of the sternum; one pair at the upper part at the manubrium, one at the middle of the sternal body and one at the sternoxiphoid junction. Each pair of crystals was oriented to detect distraction in a transverse plane. Ultrasonic gel was placed between the two crystals to facilitate sound wave conduction. The sensitivity of the distance measurement using sonomicrometry is approximately 0.02 mm. Intrathoracic pressure was increased by inflating the intrathoracic bladder using a calibrated electronic air pump until 2.0 mm of distraction was noted between the ultrasonic crystals. At the end of the experimental protocol, 1 cm^2 ^segments of mid-sternum, away from any closure instrumentation, were taken from each specimen and x-rayed in AP and lateral planes. The x-rays were reviewed and evaluated for structural differences by a radiologist blinded to group allocation.

### Data Analysis

All data were analyzed with SigmaStat (v 3.5, Systat Software San Jose, California, USA) and R (version 2.5.0, lme4) statistical programs. No differences were noted between the wire and cable methods of primary sternal closure and as such they were grouped by a priori design. A repeated measures ANOVA and the Holm-Sidak test, was preformed to discern differences between wires/cables, wires/cables + 1 plate and the 4-plate methods of closure. In addition, regression coefficients and the corresponding 95% credible interval were calculated to determine the amount of pressure the addition of one plate and the 4-plate method could withstand prior to 2 mm separation, using the wires/cables as the reference. Data is reported as mean ± standard deviation and p-value ≤ 0.05 was considered statistically significant.

## Results

The mean age of the cadavers was 80.4 years (range 45–99), and 7 were male. The analysis of the sternal bone specimens revealed similar sternal cortical thickness (mean = 1.5 mm, range 1.1–2.0 mm), with no difference between groups. The lower portion of sternal closure was the least stable part of the sternum with 65% of all separations occurring at this location, followed by the middle (12%), and the upper (4%). In 15% of the trials no crystal pair separated 2.0 mm and as such the maximum pressure attained was used for the purposes of the analysis. Two trials with the 4-plate closure technique were excluded from the analysis for technical reasons (crystal malfunction, rib fracture). There were no significant differences between the wires alone or cables alone.

**Figure 2:**
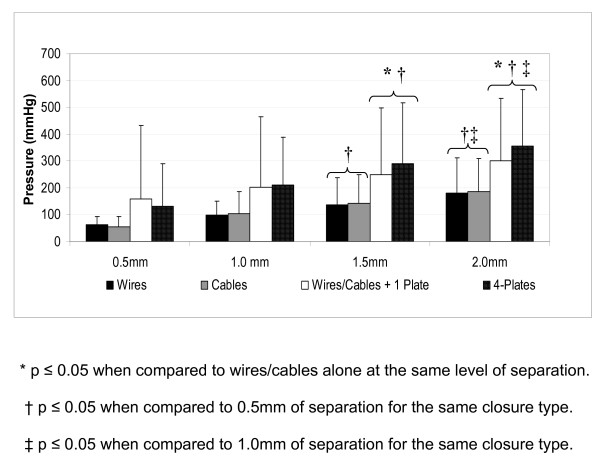
**Intrathoracic pressure for 0.5 mm incremental increases in sternal separation**. In all groups the pressure which generated 1.5 or 2.0 mm sternal separation was significantly higher than baseline or pressure for 0.5 mm separation. In addition, both plating groups required a significantly higher pressure than the wires or cables groups for either 1.5 or 2.0 mm separation.

**Figure 3:**
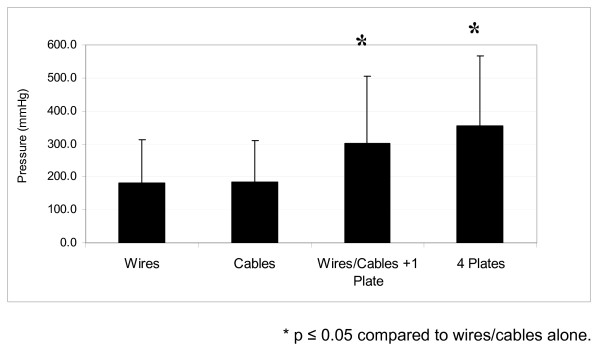
**Intrathoracic Pressure when sternal separation increased by 2.0 mm**. The mean pressure to produce 2.0 mm dehiscence in the cables or wires group was significantly lower than either the one plate or 4 plates groups (183.3 ± 123.9 vs. 301.4 ± 204.5 and 355.0 ± 210.4 respectively, p < 0.05), There was no significant difference between wires/cables plus one plate group compared to the 4 plates group.

As intrathoracic pressure increased, sternal separation increased in all cadavers as indicated by greater crystal separation. In all groups the pressure which generated 1.5 or 2.0 mm sternal separation was significantly higher than baseline or pressure for 0.5 mm separation. In addition, both plating groups required a significantly higher pressure than the wires or cables groups for either 1.5 or 2.0 mm separation (Figure 2 &3).

Data for the primary outcome of 2.0 mm sternal separation is shown in Table 1. The mean pressure to produce 2.0 mm dehiscence in the cables or wires group was significantly lower than either the one plate or 4 plates groups (183.3 ± 123.9 vs. 301.4 ± 204.5 and 355.0 ± 210.4 respectively, p < 0.05), There was no significant difference between wires/cables plus one plate group compared to the 4 plates group.

**Table 1:** Comparison of different methods of sternal closure.

Type of Sternal Closure	*Mean ± SD*	*Median (IQR)*	*Regression Coefficients (95% CI)*
*Wires* ***/*** *Cables* *(n = 18)*	183.3 ± 123.9	146.0 (122)	---

*Cables* ***/*** *Wires ****+ *** *1 Plate* *(n = 18)*	301.4 ± 204.5*	225.0 (92.0)*	120.00 (47–194) †

*4 Plates* *(n = 16)*	355.0 ± 210.4*	287.0 (242.0)*	142.00 (66–219) †

SD = Standard Deviation, IQR = Inter-quartile Range, CI = Credible Interval

* p < 0.05 compared to wires/cables alone.

† indicates statistical significance when compared to wires/cables alone

## Discussion

Sternal wound dehiscence and infections following open-heart surgery have an incidence of between 0.4–5.1%. In its minor form it is associated with a superficial wound separation and drainage which subsequently may settle. In other circumstances it can be associated with sternal pain or an abnormal clicking sensation with breathing and this is associated with loss of sternal fixation. Major complications including mediastinitis and osteomyelitis with an unacceptably high mortality rate [5]. Such complications can escalate the costs of surgery up to 4-fold. Not surprisingly, over 40 techniques have been described in the medical literature to treat sternal dehiscence with its complications, but there is no ideal method [9].

Stainless steel wire remains the most popular method for primary sternal closure because of its familiarity, simplicity and relatively low cost.

In this study we used new sternal fixation techniques; the sternal cables and the sternal plates. The sternal cable is a flexible multifilament wire that is intended to distribute the force of closure over a wider surface. Cables have a unique instrument (Tensioner) which is used to adjust the cables to an adequate tension then crimp the ferrule on top of the cable and cut it. This force is equally applied to all cables to avoid the individual force variations encountered in manual tightening of the standard wires.

The sternal plate fixation system used in this study is made of Titanium locking straight plates for sternal and rib fixation. A locking plate allows each screw to fixate to the plate distributing the forces applied to the plate to all of the screws equally facilitating a strong stable construct even when screws are inserted into softer material such as rib cartilage. This potentially improves the strength of closure by distributing the force applied at the point of closure over a larger area including the ribs on both sides. Eight screws for each plate have been recommended and were used in this study.

When compared to the standard closure technique, adding a single sternal plate to the wires or using the four plates provided significantly improved stability as indicated by the significant higher pressure required to induce sternal separation. This improvement in closure stability was consistent at each 0.5 mm measurement interval.

To avoid significant damage of the sternal bone integrity, we chose 2.0 mm as an end point of sternal distraction. We initially closed the sternum with wires or cables for the first measurement then added the single plate the plates for the second and finally, after removal of all the wires and single plate, applied the 4 plates for the final measurements. This sequence of closure was designed to mimic the clinical situation with failure of the sternal wires and even wiring with a single plate at rib 6 following which sternal pates would be applied. Despite using such a sequence that potentially biased against the 4 sternal plates due to the previous manipulations, the results of plate closure proved to be better than wires or cables alone.

Many techniques have been developed to prevent sternal dehiscence in high risk patients. Some techniques have been designed to avoid wire cutting through bone the sternal bone either by adding coils [10] or a parasternal vertically oriented basket weave wiring technique [11]. Stainless steel bands [12,13] and Mersilene ribbon [14] have also been utilized in attempts to distribute the closure force over a wider area. Sternal plates in sternal closure [15] have shown better distribution of the force across the sternum that yielding a more secure closure. Sternal plates clinically have also been used for primary closure [16]. Others [17] as well as our group have utilized sternal plates for salvage of sternal dehiscence after cardiac surgery. The advantage of these sternal plates compared to others lies on their being fixed transversely to the anterior surface of the sternum and ribs. Following surgical debridement and irrigation to healthy bleeding bone, with avoidance of dissection underneath the sternum to reduce the risk of injury to vital structures and additional ischemia to the bone, sternal edges can be approximated and the plates applied. The increase in strength with even one plate at rib 6 demonstrates its potential usefulness when localized debridement and rewiring might be considered or when wiring alone may be insufficient.

The plates come in preset lengths which can be cut and contoured to adapt to different sternal angles and rib curvature. The plates also have a release pin on each one for emergent reentry. There are also smaller star-shaped and H-shaped locking plates that facilitate fixation of bone such as the manubrium, the thickest portion of the sternum, where bone purchase is optimized. Some other devices like Pectofix plates cannot be fixed to the manubrium because of their large size [18].

There are some limitations to this study. Although fresh human cadavers were used for this model, they may not reproduce the physiological loads of respiration or coughing. Our method of applying a distraction force was also not physiologic, but it did allow for highly accurate continuous and reproducible measurements. There was variation in the cadavers leading to variable absolute measurements of sternal stability when comparing the different specimens. However, rather than using the laboratory synthetic bone or animal models, we intentionally used the human cadaver as the closest model of the real-life sternum with its variations in intrinsic strength for wiring and thickness of the bone and cartilage for plating. Interestingly, the gross and radiological appearance of the sternal bone was similar in all specimens. Improved sternal stability with the use of plating was seen in all the specimens.

## Conclusion

Sternal stability is improved with sternal plating compared to wiring techniques and can restore sternal stability after wiring. Adding a transverse plate to primary wire or cable cerclage closure substantially improves stability of sternotomy closure in a human cadaver model, and may be a consideration in high risk patients.

## Competing interests

The authors declare that they have no competing interests.

## Authors' contributions

HF conceived of the study, participated in its design, in writing the grant and carried out the coordination, running the cadaver experiments, collecting and analyzing the data, writing, reviewing and submitting the manuscript. NA participated in running the cadaver experiments. DM participated in the study design, writing the grant, running the cadaver experiments, reviewing and analyzing the data. AH participated in running the cadaver experiments and performed the statistical analysis. DL participated in writing the grant and reviewing the manuscript. DB participated in writing the grant and reviewing the manuscript. LE participated in writing the grant and reviewing the manuscript. JM conceived of the study, participated in its design, writing the grant and carried out the coordination, running the cadaver experiments, reviewing and submitting the manuscript. All authors read and approved the final manuscript.
